# In Silico and In Vitro Evaluation of the Antimicrobial and Antioxidant Potential of *Mentha* × *smithiana* R. GRAHAM Essential Oil from Western Romania

**DOI:** 10.3390/foods10040815

**Published:** 2021-04-09

**Authors:** Călin Jianu, Daniela Stoin, Ileana Cocan, Ioan David, Georgeta Pop, Alexandra Teodora Lukinich-Gruia, Marius Mioc, Alexandra Mioc, Codruța Șoica, Delia Muntean, Laura-Cristina Rusu, Ionuț Goleț, Delia Ioana Horhat

**Affiliations:** 1 Faculty of Food Engineering, Banat’s University of Agricultural Sciences and Veterinary Medicine “King Michael I of Romania” from Timisoara, Calea Aradului 119, RO-300645 Timișoara, Romania; calin.jianu@gmail.com (C.J.); danielastoin@usab-tm.ro (D.S.); ileanacocan@usab-tm.ro (I.C.); 2 Faculty of Agriculture, Banat’s University of Agricultural Sciences and Veterinary Medicine “King Michael I of Romania” from Timisoara, Calea Aradului 119, RO-300645 Timișoara, Romania; georgeta_pop@usab-tm.ro; 3 OncoGen Centre, County Hospital “Pius Branzeu”, Blvd. Liviu Rebreanu 156, RO-300736 Timisoara, Romania; gruia_alexandra@yahoo.com; 4 Faculty of Pharmacy, “Victor Babes” University of Medicine and Pharmacy, 2nd Eftimie Murgu Square, RO-300041 Timisoara, Romania; marius.mioc@umft.ro (M.M.); alexandra.mioc@umft.ro (A.M.); codrutasoica@umft.ro (C.Ș.); 5 Faculty of Medicine, “Victor Babes” University of Medicine and Pharmacy, 2nd Eftimie Murgu Square, RO-300041 Timisoara, Romania; muntean.delia@umft.ro (D.M.); horhat.ioana@umft.ro (D.I.H.); 6 Multidisciplinary Research Center on Antimicrobial Resistance, “Victor Babes” University of Medicine and Pharmacy, 2nd Eftimie Murgu Square, RO-300041 Timisoara, Romania; 7 Faculty of Dental Medicine, “Victor Babes” University of Medicine and Pharmacy, 2nd Eftimie Murgu Square, RO-300041 Timisoara, Romania; laura.rusu@umft.ro; 8 Multidisciplinary Center for Research, Evaluation, Diagnosis and Therapies in Oral Medicine, “Victor Babes” University of Medicine and Pharmacy, Spl. Tudor Vladimirescu 14A, RO-300173 Timisoara, Romania; 9 Faculty of Economics and Business Administration, West University of Timișoara, 300233 Timisoara, Romania; ionut.golet@e-uvt.ro

**Keywords:** essential oil, *Mentha* × *smithiana* R. GRAHAM, antioxidant activity, antimicrobial activity, molecular docking

## Abstract

This study was conducted to identify the volatile compounds of *Mentha* × *smithiana* essential oil (MSEO) and evaluate its antioxidant and antibacterial potential. The essential oil (EO) content was assessed by gas chromatography–mass spectrometry (GC-MS). Carvone (55.71%), limonene (18.83%), *trans*-carveol (3.54%), *cis*-carveol (2.72%), beta-bourbonene (1.94%), and caryophyllene oxide (1.59%) were the main identified compounds. The MSEO displayed broad-spectrum antibacterial effects and was also found to be the most effective antifungal agent against C*andida albicans* and *Candida parapsilosis*. The antioxidant activity of MSEO was tested against cold-pressed sunflower oil by peroxide, thiobarbituric acid, 1,1-diphenyl-2-picrylhydrazyl radical (DPPH), and β-carotene/linoleic acid bleaching methods. The EO showed strong antioxidant effects as reflected by IC_50_ values of 0.83 ± 0.01 mg/mL and relative antioxidative activity of 87.32 ± 0.03% in DPPH and β-carotene/linoleic acid bleaching assays, respectively. Moreover, in the first 8 days of the incubation period, the inhibition of primary and secondary oxidation compounds induced by the MSEO (0.3 mg/mL) was significantly stronger *(p <* 0.05) than that of butylated hydroxyanisole. In silico molecular docking studies were conducted to highlight the underlying antimicrobial mechanism as well as the in vitro antioxidant potential. Recorded data showed that the antimicrobial activity of MSEO compounds could be exerted through the D-Alanine-d-alanine ligase (DDl) inhibition and may be attributed to a cumulative effect. The most active compounds are minor components of the MSEO. Docking results also revealed that several mint EO components could exert their in vitro antioxidant activity by employing xanthine oxidase inhibition. Consequently, MSEO could be a new natural source of antioxidants and antiseptics, with potential applications in the food and pharmaceutical industries as an alternative to the utilization of synthetic additives.

## 1. Introduction

The consumption of minimally processed and additive-free foods has increased in recent decades, demanding the replacement of the traditional preservation methods by the food industry [1]. Different emerging technologies (e.g., high-pressure processing, pulsed electric field, modified atmosphere packaging) have been studied in order to prolong the shelf life of foodstuff [2], including the use of natural extracts and essential oils (EOs). EOs are aromatic, volatile, and complex liquids extracted from different plant parts (flowers, leaves, seeds, fruits, bark, roots) [3]. These are secondary metabolites mainly involved in plants’ defensive mechanisms and usually contain monoterpenes, sesquiterpene, and phenolic compounds, as well as oxygenated or non-oxygenated derivatives [4]. Aside from their multiple applications in the cosmetic, pharmaceutical, and food industry, these are also recognized for their biological properties (antimicrobial, antioxidant, carminative, antiviral, anti-inflammation, analgesic, antispasmodic, etc.) [5,6,7,8].

*Mentha* species (Lamiaceae), which includes 42 species and hundreds of subspecies, are spread worldwide, mainly in Asia, Africa, Australia, North America, and Europe [9,10,11]. Romanian flora includes about 25 species and several varieties and subspecies from the genus *Mentha* [12]. The aerial parts (e.g., leaves, flowers, and stems) of *Mentha* species have been applied for centuries in folk medicine to treat multiple dysfunctions of the gastrointestinal tract or cholecystopathies [12,13,14]. Several *Mentha* species, such as spearmint (*Mentha spicata*), peppermint (*M*. × *piperita*), and corn mint (*M. canadensis*) are extensively utilized as industrial crops for the purpose of EO production [11]. These oils have many applications as flavoring agents in chewing gums, beverages, bakery products, cosmetics, oral hygiene products, and pharmaceuticals [11,15]. The plants belonging to the *Mentha* species are mentioned as promising free radical scavengers, as well as primary antioxidants that can react with free radicals and reduce the attack of reactive oxygen species on biological and food systems [16,17,18]. Additionally, multiple investigations reported the antimicrobial and antifungal properties of the *Mentha* species EOs and/or extracts against pathogenic bacteria and fungi [3,19,20,21,22]. Nevertheless, some members of the *Mentha* genus remain partly explored, such as *Mentha* × *smithiana* R. GRAHAM, an accepted hybrid of *M. aquatica* × *M. arvensis* × *M. spicata* [23]. To the best of our knowledge, no data have been reported yet on the antioxidant properties of *Mentha* × *smithiana* essential oil (MSEO).

Therefore, this research investigated: (i) the chemical composition; (ii) the antimicrobial and antioxidant activities of MSEO; and (iii) the mechanisms of interaction between MSEO chemical compounds and target proteins associated with antibacterial effects and intracellular antioxidant mechanisms, thus aiming for its possible recommendation in food and pharmaceutic industries as a green preservative.

## 2. Materials and Methods

### 2.1. Chemicals

Anhydrous sodium sulphate, C_8_–C_20_ alkane standard mixture, hexane, chloroform, ethanol, methanol, butylated hydroxyanisole (BHA), butylated hydroxytoluene (BHT), thiobarbituric acid (TBA), 1,1-diphenyl-2-picrylhydrazyl radical (DPPH), and β-carotene were purchased from Sigma-Aldrich Chemie GmbH (Taufkirchen, Germany). All substances were used as received.

### 2.2. Essential Oil Extraction

The aerial parts (flowers, leaves, and stems) of *M. smithiana*, at the full flowering phenological stage, were manually harvested from the experimental fields of Banat’s University of Agricultural Sciences and Veterinary Medicine “King Michael I of Romania” from Timisoara (BUASVM Timisoara) in June 2019. A voucher specimen (VSNH.BUASTM-90/1) was collected, identified, and deposited in the BUASVM Timisoara Herbarium. MSEO was obtained by steam distillation, using a modified Clevenger apparatus (Corning Life Sciences, Kennebunk, ME, USA) [24], with a cooled oil collector to prevent the generation of artefacts during isolation [25]. A slightly yellow oil with a characteristic odor and sharp taste was obtained. After decantation, the water traces were removed by using anhydrous sodium sulphate, and the product was deposited at −18 °C until use.

### 2.3. Gas Chromatography–Mass Spectrometry

The MSEO was diluted 1:1000 in hexane and vortexed before injection. In total, 1 μL of the prepared sample was injected in splitless mode in a HP6890 Gas-Chromatograph coupled with a HP5973 Mass Spectrometer (Agilent Technologies, Santa Clara, CA, USA). The injected sample was run into a Bruker Br-5MS capillary column (30 m × 0.25 mm id × 0.25 μm, Bruker, Billerica, MA, USA) with helium flow of 1 mL/minute. The oven temperature ranged from 50 °C to 300 °C with 6 °C/minute rate and a final hold for 5 min; the solvent delay was 3 min. The mass spectrometer had the source set at 230 °C, the MS Quad at 150 °C, and ionization energy at 70 eV. The compounds’ mass values were scanned between 50 and 550 amu. The identification of the MSEO components was based on retention indices (RIs), calculated by means of a C_8_–C_20_ alkane standard mixture calibration curve and subsequently compared with Adams indices [26], computer matching with the NIST0.2 mass spectra library (USA National Institute of Science and Technology software, NIST, Gaithersburg, MD, USA), and by co-injection with reference samples (limonene and carvone).

### 2.4. Antioxidant Activity

In order to evaluate the antioxidant activity of the MSEO, cold-pressed sunflower oil purchased from the local market was used (1.92 meq·kg^−1^ initial peroxide value). This oil is frequently used in the South-East European countries’ cuisines [27] and is somewhat unstable due to the rich content in fatty acids [28]. The MSEO antioxidant activity was studied by peroxide, thiobarbituric acid, 1,1-diphenyl-2-picrylhydrazyl radical (DPPH), and *β*-carotene/linoleic acid bleaching tests, respectively.

#### 2.4.1. Sample Preparation

Samples were prepared by adding 0.1 mg/mL and 0.2 mg/mL, 0.3 mg/mL, respectively, of MSEO to 10 mL volume of cold-pressed sunflower oil. Additionally, 0.2 mg/mL of BHA and BHT, the maxim amount of these synthetic additives in fats and oils according to the European Union (EU) food legislation [29], were added in 10 mL cold-pressed sunflower oil. A control sample without any additive was prepared under similar conditions.

#### 2.4.2. Peroxide Value

The peroxide value (PV) of the above-prepared samples, expressed in meq of oxygen ·kg^−1^, was measured at 0, 4, 8, 12, 16, 20, and 24 days, according to ISO 27107: 2008, “Animal and vegetable fats and oils. Determination of peroxide value” [30]. All measurements were performed in triplicate.

#### 2.4.3. Thiobarbituric Acid Value

The thiobarbituric acid (TBA) test, as described by Jianu et al. [31], was adopted; briefly, 2 g of each sample, benzene (5 mL), and 0.67% aqueous thiobarbituric acid solution (4 mL) were mixed and continuously homogenized for 1 h with a mechanical shaker. Subsequently, the supernatant was maintained on a hot water bath for 45 min. After cooling, the solutions were spectrophotometrically assessed at 540 nm (Specord 210 spectrophotometer, Analytik Jena, Jena, Germany). The TBA value (μg malondialdehyde g^−1^) was measured every 4 days, and all the measurements were performed in triplicate.

#### 2.4.4. Scavenging Effect on 1,1-Diphenyl-2-picrylhydrazyl Radical (DPPH)

The scavenging effect of the MSEO on the DPPH radical was analyzed by using the adapted Brand-Williams method [32,33,34]. In total, 10 μL methanolic DPPH solution (1 mg/mL) was mixed with each analyzed sample (100 μL) at different serial concentrations (1.5 to 0.093 mg/mL). The absorbances were measured at 515 nm (Tecan i-control, 1.10.4.0 infinite 200Pro) after 30 min of incubation (in the dark at room temperature). BHT and BHA served as positive controls, while methanol was used as a negative control. The inhibition of free DPPH radical (I%) was calculated according to the following equation: I% = (A_methanol_ − A_sample_/A_methanol_) × 100, where: A_methanol_ is the absorbance of the methanol and A_sample_ is the absorbance of the tested oil. BioDataFit 1.02 program (Chang Broscience Inc., Fremont, CA, USA) was used to calculate the IC_50_. All measurements were performed in triplicate.

#### 2.4.5. β-Carotene Bleaching Assay

The analysis was conducted using the method described by Oke et al. [35], with some modifications [31]. Briefly, a β-carotene (0.5 mg) stock solution was prepared in a mixture of chloroform (1 mL), Tween 40 (200 mg), and linoleic acid (25 µL). The chloroform was removed under vacuum at 40 °C for 5 min by using a rotary evaporator (Heidolph, Schwabach, Germany). The residue was treated with 3% hydrogen peroxydeaqueous solution (100 mL) and stirred vigorously (2–3 min) to obtain an emulsion. Aliquots of the emulsion (2.5 mL) were added in the test tubes containing MSEO (350 µL); BHT was used as a positive control. All tubes were kept for 48 h at room temperature before measuring the absorbances at 490 nm. All measurements were performed in triplicate.

### 2.5. Determination of Antimicrobial Activity

#### 2.5.1. Bacterial Strains

The MSEO antibacterial and antifungal activity was tested against six Gram-positive and Gram-negative bacteria: *Salmonella enterica* serotype *Typhimurium* (ATCC 14028), *Escherichia coli* (ATCC 25922), *Pseudomonas aeruginosa* (ATCC 27853), *Shigella flexneri* serotype 2b (ATCC 12022), *Staphylococcus aureus* (ATCC 25923), and *Streptococcus pyogenes* (ATCC 19615) and two fungus strain *Candida albicans* (ATCC 10231) and *Candida parapsilosis* (ATCC 22019) (Microbiologics, France). According to the EFSA One Health 2019 Zoonoses Report, the selected microbial strains are the most frequently reported causes of foodborne outbreaks across the EU and other reporting countries [36].

#### 2.5.2. Antibacterial Activity Assay

The MSEO antimicrobial activity was assessed according to the Clinical and Laboratory Standards Institute standard [37], with some modifications [3]. A bacterial suspension of each tested strain was prepared to a concentration of 0.5 MacFarland, approximatively 1–2 × 10^8^ colony forming units (CFU)/mL for bacterial strains and 1–5 × 10^6^ CFU/mL for *Candida* strains. The Mueller-Hinton (MH) agar or Mueller-Hinton agar-fastidious organisms’ agar (MHF) supplemented with defibrinated horse blood and β-nicotinamide adenine dinucleotide (bioMérieux, Marcy-l’Étoile, France) was inoculated with 0.1 mL standardized suspension. In total, 10 µL MSEO was added to a 6 mm diameter blank paper disk (BioMaxima, Lublin, Poland) and deposited on the MH or MHF plates’ surface, respectively, previously inoculated with microbial strains. The plates were incubated for 24 h at 35–37 °C (for bacterial strains) and for 48 h at 28 °C (for yeasts), after which the diameters of the inhibitory halos (in millimeters) formed around the paper disk were measured. Gentamycin (10 µg/disk) and fluconazole (25 µg/disk) disks were used as a positive control for bacteria and yeast (BioMaxima, Lublin, Poland), respectively. Dimethylsulphoxide (DMSO) was used as the negative control. All analyses were carried out in triplicate for each microbial strain.

#### 2.5.3. Minimum Inhibitory Concentration (MIC)

MSEO strongly inhibits the growth of all tested bacterial and yeast strains; minimum inhibitory concentration (MIC) values were determined by applying microdilution in broth assay according to the Clinical and Laboratory Standards Institute (CLSI) guidelines M07-A10 for bacteria [38] and European Committee on Antimicrobial Susceptibility Testing (EUCAST) definitive document E.Def 7.2 for yeasts [39]. Standardized inoculum (0.5 McFarland meaning 10^8^ CFU/mL for bacterial strains and 2 × 10^6^ CFU/mL for fungal strains) was prepared by dilution, resulting in 1–5 × 10^5^ CFU/mL microbial suspension. Successive dilutions of MSEO in DMSO were prepared in order to achieve various concentrations (400, 200, 100, 50, 25, 12.5 mg/mL). In total, 0.1 mL of each MSEO dilution was treated with 0.4 mL MH or MHF broth and 0.5 mL microbial suspension, with a final microbial inoculum of approximately 0.5 × 10^5^ CFU/mL. After 24 h of incubation at 35–37 °C (for bacteria) and 28 °C (for yeasts), the MIC (the lowest concentration without visible growth) was assessed. As a control, 0.1 mL DMSO was added in a tube with 0.5 mL microbial suspension and 0.4 mL MH or MHF broth for bacteria and fungi, respectively. All analyses were carried out in triplicate for each microbial strain.

#### 2.5.4. Minimum Bactericidal Concentration (MBC) and Minimum Fungicidal Concentration (MFC)

The minimum bactericidal concentration (MBC) and minimum fungicidal concentration (MFC) were determined according to the method described by Danciu et al. and Jianu et al. [40,41], with some modifications. In total, 1 µL culture from each test tube at MIC (with no visible growth) was inoculated using a loop (NuovaAptaca SRL, Canelli, Italy) on Columbia agar supplemented with 5% blood and maintained for 24 h at 37 °C for bacteria or on Sabouraud supplemented with chloramphenicol medium and maintained for 48 h at 28 °C for the yeast. The MBC/MFC was established as the lowest EO concentration, which killed 99.5% of the inoculated microorganisms [42,43]. All analyses were carried out in triplicate for each microbial strain.

### 2.6. In Silico Molecular Docking

Corresponding 3D structures of the protein targets (Table 1) were obtained from the RCSB Protein Data Bank [44]. The protein structures were prepared as suitable targets, using the Autodock Tools (version 1.5.6, The Scripps Research Institute, La Jolla, CA, USA). Water molecules, undesired protein chains, metal atoms, and the co-crystalized ligands were removed from the protein structure, after which polar hydrogen atoms and Gesteiger charges were added. The target was saved as the required file format (pdbqt). Ligand molecules corresponding to the 39 MSEO components were drawn as mol files using Biovia Draw (Dassault Systems Biovia, San Diego, CA, USA) and were subsequently converted into 3D structures using PyRx’s Open Babel module. Structure geometry optimization was also achieved with Open Babel, using the ghemical force field. Compound docking was performed with the GUI software PyRx, using Autodock Vina’s scoring function [45]. In order to validate the docking method, the co-crystalized ligands were previously removed from their respective proteins, prepared as suitable pdbqt files and re-docked in their original binding sites. The predicted docking pose was compared with the experimental co-crystallized binding pose. This docking validation was performed for all protein structures. The grid box, which delimitates the search space, was defined in terms of coordinates and size (Table 1) to best fit the active binding site. Data results for docked molecules were recorded as free binding energy values (ΔG, kcal/mol). Ligand-protein binding features were analyzed using Accelerys Discovery Studio 4.1 (Dassault Systems Biovia).

### 2.7. Statistical Analysis

Statistical analysis was carried out using the SPSSv25 software (SPSS Inc., Chicago, IL, USA). In the preliminary analysis, the Shapiro–Wilk test was performed to test the normality of data for each incubation period (day 0, day 4, day 8, day 12, day 16, day 20, and day 24). Due to the non-normally found in our data, nonparametric tests were further applied. As an overall omnibus test, Kruskal–Wallis was employed to find significant differences in the case of independent samples, BHA, BHT, and three concentrations of MSEO (0.1, 0.2, and 0.3 mg/mL, respectively). The Mann–Whitney test was performed to make multiple pairwise comparisons to detect statistically significant differences between specific samples. All the above-mentioned tests were applied for each level of the incubation period. The Friedman test for dependent samples and repeated measurements was performed to analyze the time evolution over the incubation period levels. Each statistical testing procedure was applied distinctively for peroxide and TBA values. The Tukey test assessed differences among values obtained from three replicates performed in the antimicrobial analysis. For all the testing mentioned above, differences were considered significant when *p* < 0.05.

## 3. Results and Discussion

### 3.1. MSEO Chemical Composition

The steam distillation of fresh aerial parts of *M. smithiana* gave a slightly yellow EO, with an average calculated yield of 1.24 ± 0.03% (expressed as mean ± SD). The chemical composition is shown in Table 2, according to their elution order on a Br-5MS capillary column.

Thirty-nine compounds, accounting for 98.97% of total oil compositions, were identified. The major components were carvone (55.71%), limonene (18.83%), *trans*-carveol (3.54%), *cis*-carveol (2.72%), beta-bourbonene (1.94%), and caryophyllene oxide (1.59%), comprising 84.33% of the EO. A previous study, conducted in Romania, also reported carvone (72.72%) and limonene (14.66%) as the major compounds of MSEO [46]. On the other hand, Lawrence reported carvone (22.1–38.4%) together with dihydrocarvyl acetate (16.8%), beta-pinene (17.1%), 1,8-cineole (10.9%), and beta-caryophyllene (10.4%) as the main components of MSEO [23]. The yield and compositional variation of the EOs may be due to the harvesting time at different stage, storage, and extraction methods [47] and also depends on the physiological and environmental conditions (e.g., seasonal and geographical location, soil composition, climate) [11,48].

### 3.2. Assessment of Antioxidant Activity

**Figure 1:**
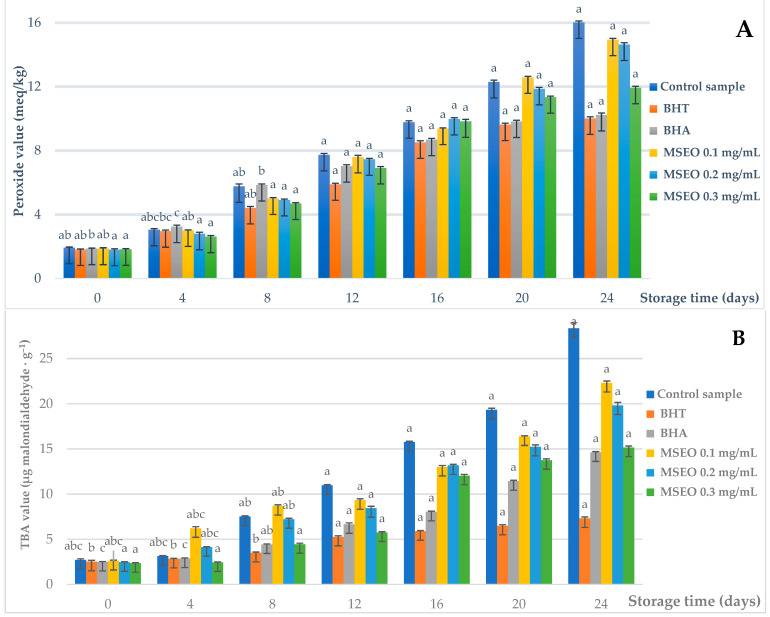
The effect of *Mentha* × *smithiana* essential oil (MSEO), butylated hydroxyanisole (BHA), and butylated hydroxytoluene (BHT) on peroxide values (PVs) (**A**) and thiobarbituric acid (TBA) values, (**B**) of cold-pressed sunflower oil during 24 days of the storage period. Values are the mean, and the error bars indicate standard deviations. Letters indicate homogenous groups within incubation periods (*p* > 0.05).

The assessment of the MSEO antioxidant activity has been conducted on cold-pressed sunflower oil. Figure 1A shows the changes in PVs of the investigated cold-pressed sunflower oil samples. The PV measures the total peroxide and hydroperoxide oxygen content of the edible oil system [49]. The Shapiro–Wilk test was applied to test the distribution of experimental data. The analysis showed that most of our data did not have a normal distribution (*p* < 0.05), except for the initial day (*p =* 0.619) and the fourth day (*p =* 0.36), which concluded the use of non-parametric tests. For each storage period time, we applied the Kruskal–Wallis test to see if we have differences between the PVs of all six tested samples. In all cases, we obtained extremely significant differences (*p <* 0.001), tested the variance of the samples using the ANOVA test, and obtained the same statistical conclusion (*p <* 0.001).

Further on, we applied the Mann–Whitney test to compare two samples at the time. The tests revealed that the PVs of the samples treated with MSEO were significantly different from those treated with BHA and BHT. One can notice that, for the initial day, the MSEO (0.2 mg/mL and 0.3 mg/mL, respectively) had significantly lower PVs compared to BHA (*p =* 0.007; *p =* 0.02). For day four, we obtained even better results for MSEO (0.2 mg/mL and 0.3 mg/mL, respectively) compared to BHT (*p <* 0.001). The same results were obtained when the MSEO (0.1, 0.2, and 0.3 mg/mL, respectively) was compared with the BHA (*p <* 0.001). On day eight, the MSEO (0.1, 0.2, and 0.3 mg/mL) had registered the same tendency versus BHA (*p <* 0.001). Moreover, we wanted to compare the treated samples’ PV variance between the storage days by using the Friedman test. The test revealed extremely significant differences (*p <* 0.001) between the time points included in the PV daily evolution study.

Peroxides are products of the primary lipid oxidation, which subsequently decomposed into carbonylic and other compounds. The peroxide decomposition products may catalyze further oxidation. TBA values of the cold-pressed sunflower oil samples were also recorded during the storage period to measure such secondary oxidation products.

The TBA value quantifies the malondialdehyde produced from unsaturated fatty acids that result from the oxidation of a lipid system [50]. Figure 1B shows the changes in the TBA values of the studied samples. The Shapiro–Wilk normality test revealed that, besides the initial day, where we had a normal distribution (*p =* 0.48), the other data were not normally distributed (*p <* 0.05). Extremely significant differences (*p <* 0.001) between the TBA values of the tested MSEO samples (0.1, 0.2, and 0.3 mg/mL, respectively) were obtained after running the Kruskal–Wallis test for each testing period. To strengthen this conclusion, we ran a variance analysis between the samples by applying an ANOVA test, which led to the same statistical conclusion (*p <* 0.001). In order to emphasize the most important differences related to this study, we tested two samples at the time by applying the Mann–Whitney test. We also analyzed the possible differences that can be measured between the TBA values of MSEO (0.1, 0.2, and 0.3 mg/mL), BHA, and BHT, respectively. On the first day, significantly lower TBA values were obtained in the case of MSEO (0.2 mg/mL and 0.3 mg/mL) compared to BHT (*p =* 0.014; *p =* 0.009) and MSEO (0.2 mg/mL and 0.3 mg/mL) compared to BHA (*p =* 0.03; *p <* 0.001). On day four, we observed extremely significantly lower TBA values in the case of MSEO (0.3 mg/mL) versus BHT and BHA (*p <* 0.001). The same tendency was noted for day eight with extremely significantly lower TBA values in the case of MSEO (0.3 mg/mL) compared to BHT (*p <* 0.001). Additionally, for each treated sample, we tested the daily evolution by applying the Friedman test, revealing extremely significant differences (*p <* 0.001).

DPPH-radical scavenging models are frequently employed in antioxidant experiments due to their suitable stability [51,52]. DPPH radicals can be scavenged based on the ability of EOs to donate hydrogen radicals to DPPH free radicals, thus reducing them to DPPH-H (2,2-diphenyl-1-picrihydrazine) [53]. The color of DPPH-H turns from purple to yellow, a process that can be quantified by measuring the absorbance at 517 nm [51]. The MSEO antioxidant activity, evaluated by the DPPH radical scavenging assay and expressed as 50% inhibition (IC_50_), is shown in Table 3. Despite the fact that the IC_50_ values of the MSEO (IC_50_: 0.83 ± 0.01 mg/mL) and BHA (IC_50_: 0.76 ± 0.01 mg/mL) are rather close, when we tested the statistical significance of their scavenging activity, extremely significant differences (*p <* 0.001) were revealed. In contrast, BHT exhibits an extremely significantly (*p <* 0.001) stronger antioxidant activity (IC_50_: 0.43 ± 0.08 mg/mL) than MSEO (Table 3). No previous investigations were reported in the literature concerning the DPPH radical scavenging capacity of MSEO. However, de Sousa Barros et al. [54] reported a similar IC_50_ value (0.86 ± 0.01 mg/mL) for the EO of *M. longifolia* (Himalayan silver mint) grown in Brasil. Compared to results reported on *M.* × *rotundifolia* from Tunisia (IC_50_: 3.77 mg/mL) [55], M. *pulegium* from Iran (IC_50_: 14.736 ± 0.156 mg/mL) [56], *M.* × *piperita* (IC_50_: 5.72 ± 0.06 mg/mL), *M. aquatica* (IC_50_: 6.75 ± 0.23 mg/mL), and *M. arvensis* (IC_50_: 57.72 ± 0.11 mg/mL) from Brasil [54], the MSEO exhibited higher activity. In contrast, EOs isolated from *M. spicata* from Tunisia (IC_50_: 10 ± 0.24 μg/mL) [19], Iran (IC_50_: 13.3 ± 0.6 μg/mL) [57], and Cyprus (IC_50_: 7.74 ± 0.20 μg/mL) [58] were more effective in scavenging DPPH free radical.

In the β-carotene and linoleic acid test, in the absence of an antioxidant, β-carotene undergoes rapid bleaching due to oxidation, which leads to the formation of free radicals. The new radicals generated by the loss of a hydrogen atom from the diallylic methylene groups attack the unsaturated β-carotene molecules, which they oxidize and partially decompose [59]. The rate of β-carotene discoloration can be decreased in the presence of antioxidants [31,60]. The relative antioxidant activity (RAA) of MSEO was calculated according to the following equation: RAA = A_MSEO_/A_BHT_, where A_BHT_ is the absorption of BHT (positive control used) and A_MSEO_ is the absorption of MSEO. Higher RAA values translated to higher antioxidant capacity. Tested through the linoleic acid system, MSEO exhibited strong antioxidant activity (87.32 ± 0.03%); however, RAA% was lower than the activity calculated for BHT (100%) (Table 3). No previous reports concerning the *β*-carotene and linoleic acid test expressed as RAA were available on the *Mentha* species’ antioxidant activity to compare the results directly.

### 3.3. Assessment of Antimicrobial Activity

The antibacterial activity was tested against eight bacterial and fungal strains (Table 4). The diameters of the inhibition halos induced by MSEO against the tested microbial strains varied between 17.66 ± 0.57 mm and 32.33 ± 2.51 mm, suggesting that the oil exerts broad-spectrum antimicrobial effects.

The results revealed that *C. albicans* and *C. parapsilosis* were the most susceptible tested strains to the MSEO action, followed by *S. pyogenes* > *S. aureus* > *E. coli* > *P. aeruginosa* > *S. flexneri* > *S. typhimurium*. Our results are in agreement with Jianu et al. [46], which reported that MSEO exerted significant antimicrobial activity against *S. aureus*, *S. typhimurium*, *P. aeruginosa*, and *C. albicans*. Furthermore, the oils extracted from other members of the mint genus (e.g., *M. longifolia, M. spicata, M. viridis, M. suaveolens*) significantly inhibited the growth of *P. aeruginosa*, *L. monocytogenes*, *C. albicans*, *S. typhimurium*, and *S. aureus* [19,20,21,22]. The recorded MICs of the tested strains were 2.5, 5, 10, and 20 mg/mL, respectively. The MBCs and MFCs were 2.5, 10, and 20 mg/mL, respectively. The MICs and MBCs were consistent with those of the inhibition zones; that is, the larger the inhibition zones’ diameter was, the smaller the MICs and MBCs would be (Table 4). Overall, the Gram-positive strains were more susceptible to the oil’s action than the Gram-negative strains, in agreement with previous investigations [61]. These susceptibility differences were presumably due to the presence of phospholipids and lipopolysaccharides in the composition of Gram-negative bacteria membrane, which provides protection against the external environment [20,62].

### 3.4. In Silico Prediction of Mechanism by Molecular Docking Analysis

Molecular docking is a powerful tool, often used to gain valuable insight into the possible molecular mechanisms of pharmacologically active substances. Herein molecular docking was employed to identify a possible mechanism of action correlated with the recorded antimicrobial effect of MSEO components. This process was used to evaluate the binding affinity of the 39 compounds from the essential oil to target proteins, usually correlated with bactericidal/bacteriostatic effects, such as DHPS, DHFR, Ddl, penicillin binding protein 1a PBP1a, DNA gyrase, type IV topoisomerase, and isoleucyl-tRNA synthetase IARS. Molecular docking was also used to assess the studied compounds’ potential to act as protein inhibitors of targets involved in intracellular antioxidant mechanisms. For this purpose, lipoxygenase, CYP2C9, NADPH-oxidase, and xanthine oxidase was used as protein targets.

The molecular docking results are shown as free binding energies (kcal/mol), the lowest values indicating a high affinity for the target protein (Table 5). The 39 compounds identified by GC/MS analysis on MSEO represent monoterpene or derived monoterpene structures, most of them having structurally similar scaffolds. Therefore, docking scores were presented as a heat map-type table, using a red-yellow color scheme, ranging from the lowest energy values, highlighted in red (in most cases corresponding to the docking score of the native ligand) to the highest, highlighted as yellow (Table 5), to easily identify a tendency of a set of compounds to act as potential inhibitor compounds with the lowest values close to that of the cocrystalized ligand) for a certain protein.

Monoterpenes are secondary plant metabolites with known antioxidant effects exerted mostly due to their conjugated double bond system within their structure. Some derivatives exhibit this effect by the additional presence of easily oxidizable groups, such as phenolic or alcoholic hydroxyls [63]. The docking results for the second subset of target proteins showed a tendency for several compounds to behave as potential inhibitors for xanthine oxidase (3NRZ). Xanthine oxidoreductase is the enzyme responsible for the catalyzed oxidation of hypoxanthine to xanthine followed by xanthine’s transformation to uric acid. In addition to this function, mammalian xanthine oxidase is a physiological source of reactive oxygen species (ROS), such as superoxide ion and hydrogen peroxide, which can function as second messengers in activating various pathways [64].

**Figure 2:**
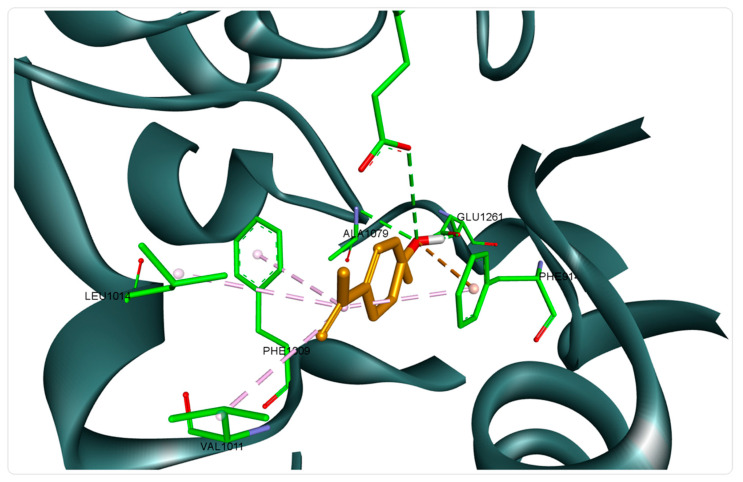
Structure of xanthine oxidase (3NRZ) in complex with *cis*-p-Mentha-2,8-dien-1-ol; hydrogen bond interactions are depicted as green dotted lines, hydrophobic interactions as purple dotted lines, and electrostatic interactions in orange; interacting amino acids are shown as green sticks.

According to the obtained docking scores, 15 of the analyzed compounds show superior affinity compared to the native cocrystallized ligand (hypoxanthine), registering free binding energies lower than the value calculated for hypoxanthine (−6.7 kcal/mol). Our predictions are in line with the findings of a previously published study, which highlighted the antioxidant potential of rich monoterpene EOs obtained from the genus *Oscimum*, assessed by HPLC-based hypoxanthine/xanthine oxidase assay [65]. Therefore, we can assume that the MSEO can exert an in vitro antioxidant effect through xanthine oxidase inhibition. The best scoring structures were the two isomers (*cis*-*trans*) of p-mentha-2,8-dien-1-ol, with the *cis* isomer recording the lowest binding energy (−7.8 kcal/mol). Binding analysis showed that the structure was well accommodated in the xanthine oxidase binding pocket through 3 HB formed with Glu802, Glu1261, and Ala1079, by means of the hydroxyl group, and was well stabilized by several additional hydrophobic interactions (Figure 2).

Regarding the set of target protein structures involved in antimicrobial activity, the results indicate an increased affinity of most docked structures towards the D-Alanine-d-alanine ligase (DDI) (2ZDQ). DDl is the enzyme that catalyzes the condensation of two D-Ala molecules using ATP to produce d-Ala-d-Ala, which is the terminal peptide of a peptidoglycan monomer. The cell-wall peptidoglycan polymer is produced through cross-linking of peptidoglycan monomer units [66]. Of the docked compounds, 17 showed similar affinity for DDl compared to that of the cocrystallized ligand (alanine, −6.2 kcal/mol) and 6 of them ((1R,4R)-4-Isopropenyl-1-methyl-2-cyclohexen-1-yl hydroperoxide, −6.6 kcal/mol; 3,6,6-Trimethyl-8-oxabicyclo [5.1.0] oct-2-en-4-one, −6.5 kcal/mol; Calamenene −6.3 kcal/mol; Cariophillene oxyde, −6.4 kcal/mol; Carvyl acetate, −6.6 kcal/mol; Lavamenthe, −6.7 kcal/mol) showed lower binding energies than those recorded for the cocrystallized ligand, indicating a possible higher affinity for the target protein. Other studies have shown that monoterpenes, such as menthol, thymol, and linalyl acetate, also exert their bactericidal effect by bacterial cell wall denaturation, causing leakage of essential nutrients [67]. Therefore, the assumption that one of the antibacterial mechanisms of the EO’s monoterpene components is the inhibition of bacterial wall synthesis, by inhibiting the DDl enzyme, is highly plausible. These results also support the fact that the antibacterial effect of the MSEO is mainly attributed to the lower concentration components. According to our results, while carvone (first major component) showed good binding affinity (−6.1 kcal/mol), slightly lower than the value calculated for Ala, limonene (second major component) is not ranked between the 17 active compounds mentioned above. In fact, the 6 compounds with the highest predicted affinity make up a little over 4% of the total oil composition.

**Figure 3:**
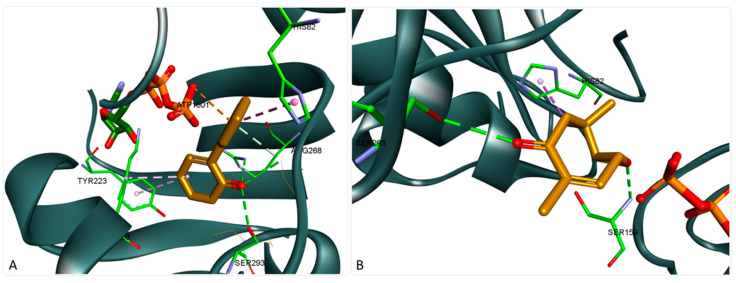
Structure of DDl (2ZDQ) in complex with lavamenthe (**A**) and 3,6,6-Trimethyl-8-oxabicyclo [5.1.0] oct-2-en-4-one (**B**); hydrogen bond interactions are depicted as green dotted lines, hydrophobic interactions as purple dotted lines, and electrostatic interactions in orange; interacting aminoacids are shown as green sticks.

Lavamenthe was recorded as the compound with the best docking score. Binding analysis showed a good accommodation of the structure within the protein binding site (Figure 3A). The oxo group is very well oriented, forming three hydrogen bonds (HBs) with Arg268, Gly288, and Ser293. The structure also interacts with ATP through an electrostatic interaction and is stabilized in the binding pocket through several hydrophobic interactions. On the other hand, compounds, such as 3,6,6-Trimethyl-8-oxabicyclo [5.1.0] oct-2-en-4-one, bearing a different scaffold than the usual monoterpene structure also recorded very good docking scores. Binding analysis showed a somewhat different interaction in the protein binding pocket (Figure 3B). The compound formed two HBs on opposite sides of the core ring, with Ser159 and Ser293, and interacted with His82 through a hydrophobic interaction but did not interact with the ATP structure.

## 4. Conclusions

Our findings revealed that MSEO is rich in monoterpene or monoterpene derivatives, mainly carvone, limonene, *trans*-carveol, *cis*-carveol, beta-bourbonene, and caryophyllene oxide. MSEO in different concentrations (0.1, 0.2, and 0.3 mg/mL, respectively) and storage period time points inhibits the formation of primary and secondary oxidation products. Moreover, the results of 1,1-diphenyl−2-picrylhydrazyl and β-carotene/linoleic acid bleaching assays indicated that the MSEO exhibits a strong antioxidant activity. The oil also exerted broad-spectrum antibacterial and antifungal effects. Molecular docking studies show that the MSEO compounds’ antimicrobial activity could be exerted due to the DDl enzyme inhibition and may be attributed to a cumulative effect; however, the most active compounds are minor components of the oil. In addition, the results suggested that the in vitro antioxidant activity presumably employs xanthine oxidase inhibition. In light of these findings, MSEO might represent a new source of natural preservatives with potential application in the food and pharmaceutical industries.

## Figures and Tables

**Table 1:** Molecular docking parameters and protein targets.

Protein	PDB ID	Grid Box Centre Coordonates	Grid Box Size	Conformers Generated per Ligand
Isoleucyl-tRNA synthetase (IARS)	1JZQ	center_x = −26.7358277569 center_y = 6.92671107775 center_z = −27.8259282538	size_x = 19.8110325702 size_y = 19.2750015157 size_z = 15.5426417959	10
DNA gyrase	1KZN	center_x = 19.4639026798 center_y = 31.387371307 center_z = 36.3586907625	size_x = 13.8319651582 size_y = 20.5700336941 size_z = 21.360339073	10
Dihydropteroate synthase (DHPS)	2VEG	center_x = 31.8624471237 center_y = 49.6265167401 center_z = 1.88555734697	size_x = 13.8319651582 size_y = 14.65456219 size_z = 14.9994242074	10
D-alanine: D-alanine ligase (Ddl1)	2ZDQ	center_x = 48.3562458265 center_y = 18.8505150195 center_z = −1.46703160733	size_x = 18.0967836094 size_y = 8.89317511714 size_z = 9.35800790526	10
Type IV topoisomerase	3RAE	center_x = −34.0399241986 center_y = 68.8943424518 center_z = −24.2150819768	size_x = 18.0967836094 size_y = 14.65456219 size_z = 18.0726700658	10
Dihydrofolate reductase (DHFR)	3SRW	center_x = −5.43716183713 center_y = −31.0341681565 center_z = 5.38290214414	size_x = 14.8382078869 size_y = 12.9264417759 size_z = 11.0341937468	10
DNA gyrase subunit B	3TTZ	center_x = 15.5996662331 center_y = −18.1561399124 center_z = 7.09296891151	size_x = 16.9958735218 size_y = 14.685112087 size_z = 12.2001752611	10
Penicillin binding protein 1a (PBP1a)	3UDI	center_x = 34.9424942577 center_y = 1.47896841514 center_z = 9.89373816917	size_x = 25.0 size_y = 12.4533701393 size_z = 21.6899971175	10
Lipoxygenase	1N8Q	center_x = 22.362960394 center_y = 1.27287112362 center_z = 20.265022301	size_x = 12.3991873959 size_y = 10.6627584168 size_z = 12.0420500164	10
CYP2C9	1OG5	center_x = −19.8236696285 center_y = 86.6979336918 center_z = 38.2757994523	size_x = 12.397236391 size_y = 11.6533632259 size_z = 11.6533632259	10
NADPH-oxidase	2CDU	center_x = 18.9974990948 center_y = −5.67040299733 center_z = −1.71861856213	size_x = 13.9673646775 size_y = 15.0103503874 size_z = 18.8052690382	10
Xanthine oxidase	3NRZ	center_x = 37.4736743805 center_y = 19.3078554887 center_z = 18.1521505909	size_x = 7.33311695257 size_y = 10.3360607773 size_z = 9.12399788674	10

**Table 2:** Components of essential oil (EO) from *M. smithiana* growing in Western Romania.

No	Compound	%	RI ^a^	Identification ^b^
1	alpha-Thujene	tr.	912	MS, RI
2	alpha-Pinene	0.97	918	MS, RI
3	Camphene	0.37	933	MS, RI
4	alpha-Phellandrene	0.45	954	MS, RI
5	beta-Pinene	0.87	959	MS, RI
6	beta-Myrcene	0.59	970	MS, RI
7	3-Octanol	0.31	976	MS, RI
8	p-Mentha-1 (7),8-diene	0.09	985	MS, RI
9	p-Cymene	0.23	1006	MS, RI
10	Limonene	18.83	1013	MS, RI, co-GC
11	Eucalyptol	0.96	1015	MS, RI
12	Terpineol, *cis*-beta	0.12	1054	MS, RI
13	Linalool	0.33	1087	MS, RI
14	Nonanal	0.06	1092	MS, RI
15	3-Octanol, acetate	0.07	1109	MS, RI
16	*trans*-p-Mentha-2,8-dien-1-ol	0.25	1111	MS, RI
17	*cis*-Limonene oxide	0.12	1124	MS, RI
18	*cis*-p-Mentha-2,8-dien-1-ol	0.47	1128	MS, RI
19	Isopinocarveol	0.08	1133	MS, RI
20	*cis*-Verbenol	0.06	1139	MS, RI
21	Menthone	0.48	1149	MS, RI
22	Borneol	0.76	1168	MS, RI
23	p-Menthan-1-ol	1.05	1175	MS, RI
24	*cis*-Dihydro carvone	0.84	1197	MS, RI
25	*cis*-Carveol	2.72	1222	MS, RI
26	*trans*-Carveol	3.54	1226	MS, RI
27	Carvone	55.71	1256	MS, RI, co-GC
28	*cis*-Carvone oxide	0.60	1284	MS, RI
29	(1R,4R)-p-Mentha-2,8-diene, 1-hydroperoxide	0.35	1332	MS, RI
30	Limonene-diol	1.07	1359	MS, RI
31	Carveol acetate	0.57	1374	MS, RI
32	Lavamenthe	0.89	1388	MS, RI
33	8-Oxabicyclo [5.1.0]oct-2-en-4-one, 3,6,6-trimethyl	0.34	1396	MS, RI
34	beta-Bourbonene	1.94	1402	MS, RI
35	*cis*-Jasmone	0.38	1409	MS, RI
36	beta-Cubebene	0.26	1450	MS, RI
37	(−)-Calamenene	0.28	1540	MS, RI
38	(−)-Spathulenol	0.37	1595	MS, RI
39	Caryophyllene oxide	1.59	1601	MS, RI
Total		98.97%		

^a^ The retention index (RI) was calculated using a homologous series of n-alkanes C_8_–C_20_; ^b^ co-GC: Co-injection with an authentic sample; tr. (trace): <0.05.

**Table 3:** Antioxidant activity of the essential oil of *M. smithiana* growing in western Romania.

Parameter	MSEO	BHA ^a^	BHT ^b^
DPPH, IC_50_ (mg/mL)	0.83 ± 0.01	0.76 ± 0.01	0.43 ± 0.08
β-carotene bleaching (RAA ^c^) (%)	87.32 ± 0.03	Nd ^d^	100

^a^ Butylated hydroxyanisole (BHA); ^b^ butylated hydroxytoluene (BHT); ^c^ relative antioxidative activity (RAA); ^d^ not detected (Nd).

**Table 4:** Antimicrobial of the essential oil of *M. smithiana* growing in Western Romania by disk diffusion, minimum inhibitory concentration (MIC), minimum bactericidal concentration (MBC), and minimum fungicidal concentration (MFC) ^1^.

Bacterial and Yeast Strains	Disk Diffusion (mm)	MIC Value (mg/mL)	MBC Value (mg/mL)	MFC Value (mg/mL)
*Streptococcus pyogenes* (ATCC 19615)	29.33 ± 0.57	5	10	N.T.
*Staphylococcus aureus* (ATCC 25923)	27.66 ± 0.57	10	10	N.T.
*Escherichia coli* (ATCC 25922)	19.66 ± 0.57	20	20	N.T.
*Salmonella typhimurium* (ATCC 14028)	17.66 ± 0.57	20	20	N.T.
*Shigella flexneri* (ATCC 12022)	18.33 ± 0.57	20	20	N.T.
*Pseudomonas aeruginosa* (ATCC 27853)	19.33 ± 2.08	20	20	N.T.
*Candida albicans* (ATCC 10231)	32.33 ± 2.51	2.5	N.T.	2.5
*Candida parapsilosis* (ATCC 22019)	31.33 ± 1.52	2.5	N.T.	2.5

^1^ The diameters of the inhibition halos are presented as the mean (*n* = 9) ± standard deviation, and the mean value for MIC, MBC, and MFC; N.T. not tested; no significant difference (*p* > 0.05) was observed by applying the Tukey test.

**Table 5:** Heat map of recorded docking scores (binding free energy—kcal/mol) of the essential oil of *M. smithiana* components ^1^.

	Protein PBD ID	1JZQ	1KZN	2VEG	2ZDQ	3RAE	3SRW	3TTZ	3UDI		1N8Q	1OG5	2CDU	3NRZ
Ligand		Binding Free Energy ∆G (kcal/mol)												
Native co-crystalized ligand		−8.3	−9.4	−6.9	−6.2	−5.6	−10.0	−8.5	−7.4		−5.8	−9.8	−9.3	−6.7
(1R,4R)-4-Isopropenyl-1-methyl-2-cyclohexen-1-yl hydroperoxide		−5.7	−6.6	−5.1	−6.6	−3.9	−6.1	−6.2	−5.6		−4.6	−6.4	−6.1	−7.4
3,6,6-Trimethyl-8-oxabicyclo[5.1.0]oct-2-en-4-one		−5.6	−5.7	−4.7	−6.5	−4.3	−6.1	−5.7	−5.2		−3.3	−5.8	−6.0	−3.1
3-octanol		−4.5	−4.6	−4.0	−4.8	−3.0	−4.7	−4.7	−3.8		−5.1	−4.8	−4.4	−5.6
3-octanyl acetate		−5.3	−5.3	−4.3	−5.4	−3.5	−5.2	−5.3	−4.4		−5.0	−5.1	−5.1	−5.3
Alpha-phellandrene		−5.8	−6.2	−4.6	−6.0	−3.6	−5.7	−5.9	−4.9		−6.0	−6.7	−6.0	−7.2
Alpha-pinene		−5.1	−5.8	−4.2	−5.0	−3.3	−5.8	−5.5	−4.6		−5.6	−6.0	−5.9	−3.3
Alpha-tujene		−5.0	−5.5	−3.8	−5.2	−3.4	−5.7	−5.3	−4.7		−6.5	−5.7	−5.6	−5.2
Beta-myrcene		−6.9	−6.4	−5.0	−3.5	−3.9	−7.6	−6.6	−5.7		−0.6	−7.3	−6.8	−1.2
Beta-pinene		−5.5	−5.0	−4.2	−5.3	−3.4	−5.3	−5.3	−4.3		−5.3	−5.5	−4.9	−6.2
Beta-bourbonene		−5.2	−6.0	−4.0	−5.4	−3.5	−5.7	−5.5	−4.6		−5.1	−5.9	−5.5	−4.5
Beta-cubebene		−6.7	−6.4	−5.1	−4.7	−4.1	−7.8	−6.8	−5.5		−4.4	−7.4	−6.8	2.1
Borneol		−5.1	−4.3	−4.3	−4.2	−3.3	−5.5	−4.7	−4.9		−2.8	−5.7	−5.3	2.0
Calamenene		−6.7	−6.2	−5.3	−6.3	−3.9	−7.6	−7.7	−6.1		−3.8	−7.6	−7.3	0.9
Camphene		−5.2	−4.6	−3.9	−4.7	−3,0	−5.4	−4.8	−4.5		−3.9	−5.7	−5.6	1.0
Cariophillene oxyde		−7.1	−6.7	−5.4	−6.4	−4.3	−8.0	−6.9	−6.1		−0.4	−7.7	−7.2	1.5
Carveol		−5.7	−6.0	−4.7	−6.0	−3.8	−5.8	−6.1	−5.1		−5.8	−6.3	−6.1	−6.9
Carvone oxide		−5.4	−5.3	−4.5	−6.0	−3.9	−5.9	−5.6	−5.6		−4.0	−5.9	−5.9	−4.6
Carvone		−5.9	−6.0	−4.8	−6.1	−3.8	−5.9	−6.0	−5.1		−5.3	−6.5	−6.2	−7.3
Carvyl acetate		−6.1	−6.8	−5.2	−6.6	−4.2	−6.5	−6.9	−5.6		−3.5	−6.7	−6.4	−5.6
*Cis*-Dihydrocarvone		−5.8	−6.0	−4.8	−6.1	−3.7	−5.9	−6.0	−5.1		−4.9	−6.4	−6.1	−7.2
*Cis*-Limonene oxide		−5.8	−6.1	−4.8	−6.0	−3.6	−5.9	−6.0	−5.0		−5.7	−6.1	−6.0	−6.7
*Cis*-jasmone		−5.6	−5.8	−4.5	−6.0	−3.5	−5.7	−5.9	−5.1		−4.4	−6.4	−5.8	−7.1
Cis-p-Mentha-2,8-dien-1-ol		−5.6	−6.3	−4.8	−6.1	−3.9	−6.0	−5.9	−5.1		−5.3	−6.3	−5.9	−7.8
*Cis*-verbenol		−5.3	−5.1	−4.3	−5.5	−3.8	−6.1	−5.4	−5.1		−4.7	−5.8	−5.7	0.3
Eucalyptol		−5.5	−4.6	−3.9	−4.9	−3.8	−5.8	−5.0	−4.8		−3.3	−5.5	−5.9	2.9
Isopinocarveol		−5.4	−4.9	−4.3	−5.4	−3.7	−6.1	−5.4	−5.3		−4.4	−5.6	−5.7	−1.3
Lavamenthe		−6.2	−6.5	−4.9	−6.7	−4.2	−6.3	−6.3	−5.8		−3.2	−6.8	−6.0	−7.1
Limonene diol		−5.6	−6.1	−5.1	−6.2	−4.1	−5.9	−6.1	−5.8		−5.2	−6.0	−6.3	−6.3
Limonene		−5.4	−5.8	−4.3	−5.6	−3.5	−5.6	−5.8	−4.6		−5.6	−6.3	−5.7	−6.8
Linalool		−5.4	−5.4	−4.3	−5.7	−3.4	−5.7	−5.9	−4.6		−4.8	−5.5	−5.0	−5.0
Menthone		−5.2	−5.7	−4.3	−5.8	−3.9	−5.8	−5.8	−5.1		−4.6	−6.3	−5.6	−7.0
Nonanal		−4.4	−4.8	−4.0	−4.5	−3.0	−4.7	−4.9	−3.9		−4.9	−4.9	−4.7	−5.7
P-cymene		−5.5	−5.8	−4.5	−5.7	−3.5	−5.6	−5.7	−4.7		−6.0	−6.2	−5.7	−6.9
P-Mentha-1(7),8-diene		−5.4	−5.8	−4.4	−5.6	−3.5	−5.6	−5.8	−4.6		−5.5	−6.3	−5.7	−6.8
P-menthan-1-ol		−5.5	−6.2	−4.7	−5.9	−3.7	−5.7	−5.8	−5.1		−4.0	−6.1	−5.8	−7.2
Spathulenol		−6.8	−6.4	−5.4	−5.5	−4.3	−8.0	−7.1	−6.0		−1.2	−7.9	−7.1	4.5
Terpineol, *cis*-beta		−5.6	−6.2	−4.7	−6.0	−3.8	−5.7	−5.8	−5.1		−4.1	−6.2	−5.8	−7.4
*Trans*-p-Mentha-2,8-dien-1-ol		−5.7	−6.2	−4.7	−6.0	−3.8	−5.7	−5.8	−5.1		−4.4	−6.3	−5.8	−7.6

^1^ Color scale varies from red to yellow (lowest recorded binding free energy to highest). First subset (left) corresponds to targets involved in antimicrobial activity, while the second subset (right) corresponds to proteins involved in antioxidant activity.

## References

[1] Barba F.J., Terefe N.S., Buckow R., Knorr D., Orlien V. (2015). New opportunities and perspectives of high pressure treatment to improve health and safety attributes of foods. A review. Food Res. Int..

[2] Ephrem E., Najjar A., Charcosset C., Greige-Gerges H. (2018). Encapsulation of natural active compounds, enzymes, and probiotics for fruit juice fortification, preservation, and processing: An overview. J. Funct. Foods.

[3] Muntean D., Licker M., Alexa E., Popescu I., Jianu C., Buda V., Dehelean C.A., Ghiulai R., Horhat F., Horhat D. (2019). Evaluation of essential oil obtained from *Mentha×piperita* L. against multidrug-resistant strains. Infect. Drug Resist..

[4] Dhifi W., Bellili S., Jazi S., Bahloul N., Mnif W. (2016). Essential oils’ chemical characterization and investigation of some biological activities: A critical review. Medicines.

[5] Bhagat M., Sangral M., Kumar A., Rather R.A., Arya K. (2020). Chemical, biological and in silico assessment of *Ocimum viride* essential oil. Heliyon.

[6] Blowman K., Magalhães M., Lemos M., Cabral C., Pires I. (2018). Anticancer properties of essential oils and other natural products. Evid.-Based Complementary Altern. Med..

[7] Bhagat M., Sangral M., Arya K., Rather R.A. (2018). Chemical characterization, biological assessment and molecular docking studies of essential oil of *Ocimum viride* for potential antimicrobial and anticancer activities. BioRxiv.

[8] Govindarajan M., Vaseeharan B., Alharbi N.S., Kadaikunnan S., Khaled J.M., Al-Anbr M.N., Alyahya S.A., Maggi F., Benelli G. (2018). High efficacy of (Z)-γ-bisabolene from the essential oil of *Galinsoga parviflora* (Asteraceae) as larvicide and oviposition deterrent against six mosquito vectors. Environ. Sci. Pollut. Res..

[9] Dorman H.D., Koşar M., Kahlos K., Holm Y., Hiltunen R. (2003). Antioxidant properties and composition of aqueous extracts from *Mentha* species, hybrids, varieties, and cultivars. J. Agric. Food Chem..

[10] Salehi B., Stojanović-Radić Z., Matejić J., Sharopov F., Antolak H., Kręgiel D., Sen S., Sharifi-Rad M., Acharya K., Sharifi-Rad R. (2018). Plants of genus *Mentha*: From farm to food factory. Plants.

[11] Singh P., Pandey A.K. (2018). Prospective of essential oils of the genus *Mentha* as biopesticides: A Review. Front. Plant Sci..

[12] Ciocârlan V. (2009). Flora ilustrată a României: Pteridophyta et Spermatophyta.

[13] Mimica-Dukic N., Bozin B. (2008). *Mentha* L. species (Lamiaceae) as promising sources of bioactive secondary metabolites. Curr. Pharm. Des..

[14] Mogosan C., Vostinaru O., Oprean R., Heghes C., Filip L., Balica G., Moldovan R.I. (2017). A comparative analysis of the chemical composition, anti-inflammatory, and antinociceptive effects of the essential oils from three species of *Mentha* cultivated in Romania. Molecules.

[15] Shahidi F., Ambigaipalan P. (2015). Phenolics and polyphenolics in foods, beverages and spices: Antioxidant activity and health effects—A review. J. Funct. Foods.

[16] Benabdallah A., Boumendjel M., Aissi O., Rahmoune C., Boussaid M., Messaoud C. (2018). Chemical composition, antioxidant activity and acetylcholinesterase inhibitory of wild *Mentha* species from northeastern Algeria. S. Afr. J. Bot..

[17] Bouyahya A., Lagrouh F., El Omari N., Bourais I., El Jemli M., Marmouzi I., Salhi N., Faouzi M.E.A., Belmehdi O., Dakka N. (2020). Essential oils of *Mentha viridis* rich phenolic compounds show important antioxidant, antidiabetic, dermatoprotective, antidermatophyte and antibacterial properties. Biocatal. Agric. Biotechnol..

[18] Nikavar B., Ali N.A., Kamalnezhad M. (2008). Evaluation of the antioxidant properties of five *Mentha* species. Iran. J. Pharm. Sci..

[19] Dhifi W., Jelali N., Mnif W., Litaiem M., Hamdi N. (2013). Chemical composition of the essential oil of *Mentha spicata* L. from Tunisia and its biological activities. J. Food Biochem..

[20] Benali T., Bouyahya A., Habbadi K., Zengin G., Khabbach A., Achbani E.H., Hammani K. (2020). Chemical composition and antibacterial activity of the essential oil and extracts of *Cistus ladaniferus* subsp. ladanifer and *Mentha suaveolens* against phytopathogenic bacteria and their ecofriendly management of phytopathogenic bacteria. Biocatal. Agric. Biotechnol..

[21] Mohkami Z., Ranjbar A., Bidarnamani F. (2014). Essential oil compositions and antibacterial properties of mint (*Mentha longifolia* L.) and rosemary (*Rosmarinus officinalis*). Annu. Res. Rev. Biol..

[22] Nikšić H., Bešović E.K., Makarević E., Durić K. (2012). Chemical composition, antimicrobial and antioxidant properties of *Mentha longifolia* (L.) Huds. essential oil. J. Health Sci..

[23] Lawrence B.M. (2006). Mint: The genus Mentha.

[24] Craveiro A.A. (1981). Óleos Essenciais de Plantas do Nordeste.

[25] Stahl E. (1984). Das ätherische Öl aus *Thymus praecox* ssp. arcticus isländischer Herkunft. Planta Med..

[26] Adams R.P. (2007). Identification of Essential oil Components by Gas Chromatography/Mass Spectrometry.

[27] Kostadinović Veličkovska S., Brühl L., Mitrev S., Mirhosseini H., Matthäus B. (2015). Quality evaluation of cold-pressed edible oils from Macedonia. Eur. J. Lipid Sci. Technol..

[28] Konuskan D.B., Arslan M., Oksuz A. (2019). Physicochemical properties of cold pressed sunflower, peanut, rapeseed, mustard and olive oils grown in the Eastern Mediterranean region. Saudi J. Biol. Sci..

[29] European Commission (2006). Directive 2006/52/EC of the European Parliament and of the Council of 5 July 2006 amending Directive 95/2/EC on food additives other than colours and sweeteners and Directive 94/35/EC on sweeteners for use in foodstuffs. O. J. Eur. Union.

[30] ISO (2008). Animal and Vegetable Fats and Oils-Determination of Peroxide Value-Potentiometric End-Point Determination.

[31] Jianu C., Goleț I., Stoin D., Cocan I., Lukinich-Gruia A.T. (2020). Antioxidant activity of *Pastinaca sativa* L. ssp. sylvestris [Mill.] Rouy and Camus essential oil. Molecules.

[32] Brand-Williams W., Cuvelier M.E., Berset C. (1995). Use of a free radical method to evaluate antioxidant activity. LWT Food Sci. Technol..

[33] Jianu C., Mihail R., Muntean S.G., Pop G., Daliborca C.V., Horhat F.G., Nitu R. (2015). Composition and antioxidant capacity of essential oils obtained from *Thymus vulgaris*, *Thymus pannonicus* and *Satureja montana* grown in Western Romania. Rev. Chim..

[34] Jianu C., Mişcă C., Muntean S.G., Gruia A.T. (2015). Composition, antioxidant and antimicrobial activity of the essential oil of *Achillea collina* Becker growing wild in western Romania. Hem. Ind..

[35] Oke F., Aslim B., Ozturk S., Altundag S. (2009). Essential oil composition, antimicrobial and antioxidant activities of *Satureja cuneifolia* Ten. Food Chem..

[36] EFSA (2021). The European Union one health 2019 zoonoses report. Efsa J..

[37] Clinical and Laboratory Standards Institute (2015). Performance Standards for Antimicrobial Disk Susceptibility Tests.

[38] Clinical and Laboratory Standards Institute (2015). Methods for Dilution Antimicrobial Susceptibility Tests for Bacteria That Grow Aerobically.

[39] Rodriguez-Tudela J.L., Arendrup M.C., Barchiesi F., Bille J., Chryssanthou E., Cuenca-Estrella M., Dannaoui E., Denning D.W., Donnelly J.P., Dromer F. (2008). EUCAST Definitive Document EDef 7.1: Method for the determination of broth dilution MICs of antifungal agents for fermentative yeasts: Subcommittee on Antifungal Susceptibility Testing (AFST) of the ESCMID European Committee for Antimicrobial Susceptibility Testing (EUCAST)∗. Clin. Microbiol. Infect..

[40] Danciu C., Muntean D., Alexa E., Farcas C., Oprean C., Zupko I., Bor A., Minda D., Proks M., Buda V. (2019). Phytochemical characterization and evaluation of the antimicrobial, antiproliferative and pro-apoptotic potential of *Ephedra alata* Decne. hydroalcoholic extract against the MCF-7 breast cancer cell line. Molecules.

[41] Jianu C., Moleriu R., Stoin D., Cocan I., Bujancă G., Pop G., Lukinich-Gruia A.T., Muntean D., Rusu L.-C., Horhat D.I. (2021). Antioxidant and antibacterial activity of *Nepeta×faassenii* Bergmans ex Stearn essential oil. Appl. Sci..

[42] Brezoiu A.-M., Prundeanu M., Berger D., Deaconu M., Matei C., Oprea O., Vasile E., Negreanu-Pîrjol T., Muntean D., Danciu C. (2020). Properties of *Salvia officinalis* L. and *Thymus serpyllum* L. Extracts Free and Embedded into Mesopores of Silica and Titania Nanomaterials. Nanomaterials.

[43] Nikolić M., Marković T., Mojović M., Pejin B., Savić A., Perić T., Marković D., Stević T., Soković M. (2013). Chemical composition and biological activity of *Gaultheria procumbens* L. essential oil. Ind. Crop Prod..

[44] Berman H.M., Westbrook J., Feng Z., Gilliland G., Bhat T.N., Weissig H., Shindyalov I.N., Bourne P.E. (2000). The protein data bank. Nucleic Acids Res..

[45] Trott O., Olson A.J. (2010). AutoDock Vina: Improving the speed and accuracy of docking with a new scoring function, efficient optimization, and multithreading. J. Comput. Chem..

[46] Jianu C., Golet I., Misca C., Jianu A.M., Pop G., Gruia A.T. (2016). Antimicrobial properties and chemical composition of essential oils isolated from six medicinal plants grown in Romania against foodborne pathogens. Rev. Chim.(Buchar.).

[47] Rohloff J., Dragland S., Mordal R., Iversen T.-H. (2005). Effect of harvest time and drying method on biomass production, essential oil yield, and quality of peppermint (*Mentha*×*piperita* L.). J. Agric. Food Chem..

[48] Figueiredo A.C., Barroso J.G., Pedro L.G., Scheffer J.J. (2008). Factors affecting secondary metabolite production in plants: Volatile components and essential oils. Flavour Fragr. J..

[49] Singh G., Kapoor I., Singh P., de Heluani C.S., de Lampasona M.P., Catalan C.A. (2008). Chemistry, antioxidant and antimicrobial investigations on essential oil and oleoresins of *Zingiber officinale*. Food Chem. Toxicol..

[50] Domínguez R., Pateiro M., Gagaoua M., Barba F.J., Zhang W., Lorenzo J.M. (2019). A comprehensive review on lipid oxidation in meat and meat products. Antioxidants.

[51] Lu C., Li H., Li C., Chen B., Shen Y. (2018). Chemical composition and radical scavenging activity of *Amygdalus pedunculata* Pall leaves’ essential oil. Food Chem. Toxicol..

[52] Ma Y.-L., Zhu D.-Y., Thakur K., Wang C.-H., Wang H., Ren Y.-F., Zhang J.-G., Wei Z.-J. (2018). Antioxidant and antibacterial evaluation of polysaccharides sequentially extracted from onion (*Allium cepa* L.). Int. J. Biol. Macromol..

[53] Molyneux P. (2004). The use of the stable free radical diphenylpicrylhydrazyl (DPPH) for estimating antioxidant activity. Songklanakarin J. Sci. Technol..

[54] de Sousa Barros A., de Morais S.M., Ferreira P.A.T., Vieira Í.G.P., Craveiro A.A., dos Santos Fontenelle R.O., de Menezes J.E.S.A., da Silva F.W.F., de Sousa H.A. (2015). Chemical composition and functional properties of essential oils from *Mentha* species. Ind. Crop Prod..

[55] Ben Haj Yahia I., Jaouadi R., Trimech R., Boussaid M., Zaouali Y. (2019). Variation of chemical composition and antioxidant activity of essential oils of *Mentha* x *rotundifolia* (L.) Huds. (Lamiaceae) collected from different bioclimatic areas of Tunisia. Biochem. Syst. Ecol..

[56] Kamkar A., Javan A.J., Asadi F., Kamalinejad M. (2010). The antioxidative effect of Iranian *Mentha pulegium* extracts and essential oil in sunflower oil. Food Chem. Toxicol..

[57] Hussain A.I., Anwar F., Shahid M., Ashraf M., Przybylski R. (2010). Chemical composition, and antioxidant and antimicrobial activities of essential oil of spearmint (*Mentha spicata* L.) from Pakistan. J. Essent Oil Res..

[58] Chrysargyris A., Xylia P., Botsaris G., Tzortzakis N. (2017). Antioxidant and antibacterial activities, mineral and essential oil composition of spearmint (*Mentha spicata* L.) affected by the potassium levels. Ind. Crop Prod..

[59] Amiri H. (2010). Antioxidant activity of the essential oil and methanolic extract of *Teucrium orientale* (L.) subsp. taylori (Boiss.) Rech. f. Iran. J. Pharm. Res..

[60] Kulisic T., Radonic A., Katalinic V., Milos M. (2004). Use of different methods for testing antioxidative activity of oregano essential oil. Food Chem..

[61] Soković M., Marin P., Brkić D., van Griensven L.J. (2008). Chemical composition and antibacterial activity of essential oils of ten aromatic plants against human pathogenic bacteria. Food.

[62] Saunders N.A., Lee M.A. (2013). Real-Time PCR: Advanced Technologies and Applications.

[63] Wojtunik-Kulesza K.A., Kasprzak K., Oniszczuk T., Oniszczuk A. (2019). Natural monoterpenes: Much more than only a scent. Chem. Biodivers..

[64] Battelli M.G., Polito L., Bortolotti M., Bolognesi A. (2015). Xanthine oxidoreductase-derived reactive species: Physiological and pathological effects. Oxid. Med. Cell. Longev..

[65] Salles Trevisan M.T., Vasconcelos Silva M.G., Pfundstein B., Spiegelhalder B., Owen R.W. (2006). Characterization of the volatile pattern and antioxidant capacity of essential oils from different species of the genus Ocimum. J. Agric. Food Chem..

[66] Kitamura Y., Ebihara A., Agari Y., Shinkai A., Hirotsu K., Kuramitsu S. (2009). Structure of d-alanine-d-alanine ligase from Thermus thermophilus HB8: Cumulative conformational change and enzyme–ligand interactions. Acta Crystallogr. Sect. D Biol. Crystallogr..

[67] Trombetta D., Castelli F., Sarpietro M.G., Venuti V., Cristani M., Daniele C., Saija A., Mazzanti G., Bisignano G. (2005). Mechanisms of antibacterial action of three monoterpenes. Antimicrob. Agents Chemother..

